# Study on the Flame Retardancy and Hazard Evaluation of Poly(acrylonitrile-co-vinylidene chloride) Fibers by the Addition of Antimony-Based Flame Retardants

**DOI:** 10.3390/polym14010042

**Published:** 2021-12-23

**Authors:** Hyelim Kim, Ji-Su Kim, Wonyoung Jeong

**Affiliations:** 1 Material and Component Convergence R&D Department, KITECH, Ansan 15588, Korea; hyelim1221@kitech.re.kr; 2 Advanced Testing & Evaluation Center, FITI Testing & Research Institute, Seoul 07791, Korea; jisu123@fitiglobal.com

**Keywords:** flame retardancy, poly(acrylonitrile-co-vinylidene chloride), antimony trioxide, antimony tetroxide, hazard evaluation

## Abstract

Antimony oxide (ATO) is used mainly as a flame retardant, but it is classified as a hazardous substance. Therefore, regulations on the use of antimony trioxide (ATO(3)) and antimony pentoxide (ATO(5)) in textile products are being developed. Accordingly, there is a need for alternative flame retardants. In this study, antimony tetroxide (ATO(4)), which has higher thermal stability and resistance to acids and alkalis than ATO(3) or ATO(5), was selected to assess its use as an alternative flame retardant. First, ATO(3) or ATO(4) were added to poly(acrylonitrile-co-vinylidene chloride) (PANVDC), and the film and wet-spun fiber were prepared. The PANVDC film with flame retardants was prepared to evaluate the flame retardancy and the mechanism of action of the flame retardants. Flame retardancy analysis showed that a limiting oxygen index of 31.2% was obtained when ATO(4) was added, which was higher than when ATO(3) was used. Subsequently, PANVDC fibers with antimony oxide were manufactured and showed improved mechanical and thermal properties when ATO(4) was used, compared to when ATO(3) was tested. In addition, migration analysis due to antimony in the fiber confirmed that the elution amount was below the acceptable standard when PANVDC fibers with ATO(4) were added. Therefore, based on these results, the flame-retardant and thermal properties of antimony tetroxide were superior to antimony trioxide, and it was confirmed that ATO(4) could be used as an alternative flame retardant to ATO(3).

## 1. Introduction

As fire-related accidents increase in industry worldwide, the demand for flame-retardant (FR) materials and work clothes related directly to safety is continuously increasing every year. Accordingly, the demand for FR fabrics that are comfortable to wear and lightweight is increasing. Currently, modacrylic fibers, which have excellent flame retardancy, price competitiveness, and an excellent tactile feel, are attracting attention as a FR workwear fiber material. Modacrylic fibers are defined as those fibers containing 35–85 wt.% acrylonitrile (AN) units. Poly(acrylonitrile-co-vinylidene chloride) (PANVDC) fibers are copolymers synthesized for modacrylic fibers [1,2,3]. Generally, poly(acrylonitrile) (PAN) has a limiting oxygen index (LOI) in the range of 18–20%, while modacrylic fibers have excellent flame retardant properties in the range of 26–31% LOI [4,5,6]. On the other hand, various flame retardant additives are used to maximize the LOI because a higher level of flame retardancy is required in extreme environments at high temperatures [7,8,9].

Antimony oxide is one of the most representative flame retardants. Antimony trioxide (ATO, Sb_2_O_3_) is commonly used with halogenated flame retardants to yield a promotional effect. The material does not have excellent flame retardancy itself, but when applied to halogen-based compounds, such as poly(vinyl chloride) (PVC), it works in the gas phase by facilitating the migration of halogens and antimony into the gas phase for flame retardation/inhibition [3,7,8,10,11]. Accordingly, the incorporation of ATO in poly(acrylonitrile-co-vinyl chloride) (PANVC) or poly(acrylonitrile-co-vinylidene chloride) (PANVDC) fibers has synergistic effects on their flame retardancy. On the other hand, antimony trioxide was recently classified as a carcinogen in Europe, and its use was regulated [12,13]. Therefore, various studies were conducted to reduce the antimony oxide used for halogen compounds and use a human-friendly flame retardant additive that could replace it [9,14]. In a previous study, a flame retardancy was confirmed by adding a different type of flame retardant to replace antimony trioxide, and, as a result, it was reported that flame retardancy was indicated even when a small amount of antimony trioxide was mixed or entirely replaced [15,16,17,18]. On the other hand, research on new flame-retardant additives is needed because it is difficult to sufficiently reduce the use of antimony trioxide.

Antimony tetroxide (Sb_2_O_4_) contains a mixed valance of Sb_2_O_3_ and Sb_2_O_5_ [11]. Although antimony tetraoxide (Sb2O4) is an antimony-based compound, as is antimony trioxide (Sb_2_O_3_), there are few reports on its toxicity, flame retardancy or antimony elution when applied to textiles. Therefore, research in this area is required. In the present study, antimony tetroxide was selected as an alternative flame retardant additive to antimony trioxide, which is generally used in modacrylic fibers. Each additive was added to poly(acrylonitrile-*co*-vinylidene chloride) to prepare the fibers through a wet-spinning process. To investigate their physical properties, the thermal and flame retardancy of the prepared spun fibers were evaluated, and the antimony elution in acidic and alkaline solutions was analyzed.

## 2. Experimental Section

### 2.1. Materials

**Figure 1:**
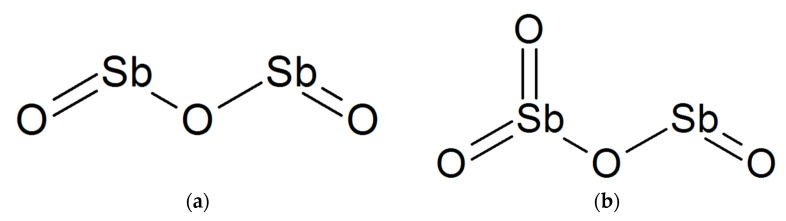
Molecular structure of the antimony oxides (**a**) Antimony trioxide (ATO(3)), (**b**) Antimony tetroxide (ATO(4)).

The poly (acrylonitrile-co-vinylidene chloride) (PANVDC) copolymer was used in this study. The PANVDC is a commercial flame-retardant modacrylic material with a composition of acrylonitrile (AN, Across, ≥99%) and vinylidene chloride (VDC, Across, ≥98.0%) and a molar ratio of 7:3. The number average molecular weight and polydispersity of PANVDC were 62,000 g/mol and 2.56, respectively. Figure 1 shows the two flame retardant additives used in this study, Antimony trioxide (Sb_2_O_3_, ATO(3)) and antimony tetroxide (Sb_2_O_4_, ATO(4)), which were purchased from Sooyangchemtec Co. Ltd., (Yesan, Korea). Dimethyl sulfoxide (DMSO, 99.9%, Samchun Chemical Co., Ltd., Seoul, Korea) was used as the solvent without further purification. 

### 2.2. Wet Spinning of PANVDC Flame-Retardant Fibers

**Figure 2:**
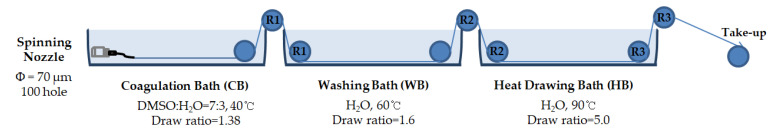
Wet-spinning system used in this study.

Figure 2 represents the scheme of wet-spinning system. The PANVDC fibers, along with the types of flame retardants, were prepared using a laboratory-scale wet-spinning system [16]. DMSO was used as the solvent because it is relatively less toxic than other solvents. To prepare the spinning solution, 35 wt.% of PANVDC was dissolved in DMSO. The flame retardant, either Sb_2_O_3_ (ATO(3)) or Sb_2_O_4_ (ATO(4)), was dispersed using an ultrasonic bath (Power sonic 420, Whashin Tech Co., Gyeonggi, South Korea) for 20 min. For the wet-spinning process, the prepared spinning solution was stored at 50 °C, and filtered through a 400 mesh, and spun into a DMSO/H_2_O coagulation bath (70/30 *w*/*w*) using a 100-filament spinneret with 70 μm capillary diameters at a speed of 1.22 mL/min. Distilled water was used for the washing bath and heat drawing bath. Subsequently, the residual solvent was prepared by drawing and drying processes. The fibers were finally dried in a 60 °C convection oven for 24 h. 

### 2.3. Characterization

- Flame-Retardant Mechanisms

Pyrolysis–gas chromatography-mass spectrometry (Py-GC/MS) was investigated using the sampling procedure reported elsewhere [16]. Py-GC/MS was conducted on a pyroprobe (PY-2020iD, Frontier, Fukushima, Japan) combined with a gas chromatograph (7890, Agilent, Santa Clara, CA, USA) and mass spectrometer (5975, Agilent, Santa, Clara, CA, USA). The pyrolysis temperature ranged from room temperature to 600 °C in a nitrogen atmosphere at a heating rate of 20 °C/min. The volatile products were then sent to the GC injector with a set temperature of 320 °C. The char layer of the PANVDC fibers was examined by scanning electron microscopy combined with energy-dispersive X-ray spectroscopy (SEM-EDX, SU8000, Hitachi, Tokyo, Japan).

- Flame Retardancy of PANVDC Film

Flame retardancy was investigated by performing two tests reported elsewhere [16,17]. All samples were prepared in the form of a film. First, the vertical burning tests were conducted according to the UL 94 (UnderWriter’s Laboratory, Vertical Burning Test) under controlled laboratory conditions. The test was performed twice with 20 mm vertical flame contacting a 125 mm × 13 mm molded sample for 10 s. To pass UL 94 V2, the flame should extinguish within 30 s after each ignition. Burning drips are allowed. For UL 94 V0, the flame should extinguish within 10 s after each ignition, with less than 50 s for the total burning of five samples and no burning drips. Another method used to analyze the flame retardancy in this study was the LOI, which is the standard of ISO 4589-2. At this time, the size of the specimen was 100 mm × 10 mm × 0.05 mm.

- Morphology and Mechanical properties of PANVDC Fiber

The morphology of the PANVDC fibers containing the flame retardant was investigated by analyzing a cross-section of a prepared fiber by field emission scanning electron microscopy (FE-SEM, Hitachi SU8000, Hitachi High-Technologies Corporation, Tokyo, Japan). To confirm the mechanical properties of each sample, the tensile properties of single fibers were tested using the Favimat fiber test system (Favimat fiber test system2, Textehchno H., Mönchengladbach, Germany). The test was conducted at least 10 times at a crosshead speed of 20 mm/min and a gauge length of 20 mm.

- Thermal Properties of PANVDC Fiber

Thermogravimetric analysis (TGA, TA Instruments Q 500, New Castle, DE, USA) was performed under flowing air at a heating rate of 20 °C/min from room temperature to 800 °C.

- Migration of antimony in the PANVDC fibers

**Figure 3:**
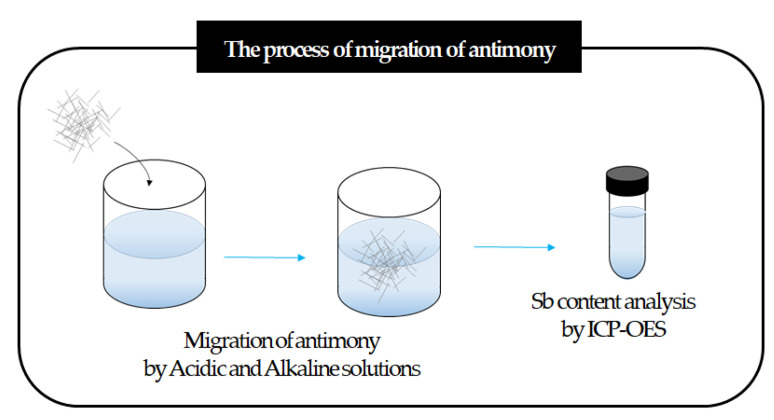
Process of migration of antimony.

According to the standard of KS G ISO 8124-3 and KS K 0731, antimony elution was evaluated in acidic and alkaline solutions, such as sweat and saliva. Figure 3 shows the process of migration of antimony. First, impregnating a 100 mg fiber specimen in the test solution. Using that solution, centrifugation was performed to separate the specimen and the eluate. After that, antimony contents in this solution were investigated through inductively coupled plasma–optical emission spectroscopy (ICP-OES, ULTIMA 2, HORIBA Scientific, Kyoto, Japan). The evaluation was performed after stabilizing the eluate concentration to 1 mol/L for storage for more than one day.

The extractable antimony content in the sample was calculated using Equation (1):

(*C* − *C*_0_)/*W* × *V* × *F*, (1)

where *C* is the concentration of the object element in the test solution (mg/L); *C*_0_ is the concentration of target element among background test solution (mg/L); *W* is mass of the specimen (g); *V* is the volume of test solution used (mL); and *F* is the dilution rate. 

## 3. Results and Discussion

### 3.1. Flame Retardant Mechanism of PANVDC Films with Flame Retardants

Table 1 lists the Py-GC/MS results of PANVDC fibers with either ATO(3) or ATO(4). Py-GC/MS of evolved gases of the PANVDC fibers at thermal decomposition at 600 °C was carried out. Sb_2_O_4_ is a compound of Sb_2_O_3_ and Sb_2_O_5_, containing a mixed valence of both materials. The density of Sb_2_O_4_ (307.52 g/mol, 6.64 g/cm^3^) is higher than Sb_2_O_3_ (291.52 g/mol, 5.20 g/cm^3^) and Sb_2_O_5_ (323.52 g/mol, 3.78 g/cm^3^), but its molecular weight is intermediate. In addition, the two stable modifications of Sb_2_O_4_ are the room temperature orthorhombic α-phase (cervantite) and the high-temperature monoclinic β-phase [11]. 

As shown in Table 1, the major pyrolysis products of the PANVDC-based fibers included hydrogen chloride (HCl). Here, HCl plays a vital role in the flame retardancy of halogen compounds and the synergistic effect of hydrogen chloride and antimony compounds in the flame-retardant mechanisms of halogen compounds [7]. Compared to PANVDC and PANVDC-ATO(3), the decomposition compounds were the same except for the antimony compounds. Pyridine, which is the decomposition compound of the chloropyridine isomer, found in both PANVDC and PANVDC-ATO (3), appears to be caused by the formation of stable carbonized structures in polyacrylonitrile (PAN) [16,19].

An antimony compound was detected as a volatile thermal decomposition product, confirming that both ATO(3) and ATO(4) had a flame retardant mechanism in the gas phases. On the other hand, in the case of PANVDC-ATO(4), cyanopentadiene, 3-methylbenzonitrile, and 3-chlorobenzonitrile, including chloropyridine isomers, which are identified as the major peak in PANVDC and PANVDC-ATO(3), were not detected. When PANVDC contained ATO(4), it exhibited a flame retardancy in a reaction different from PANVDC-ATO(3). According to the properties of antimony oxides, ATO(4) does not exhibit a significant change in the carbon–nitrogen bond of the polycyclic structure after thermal decomposition at 600 °C when added to PANVDC fibers because ATO(4) has a stable structure and exhibits a heat resistance at high temperatures. 

**Figure 4:**
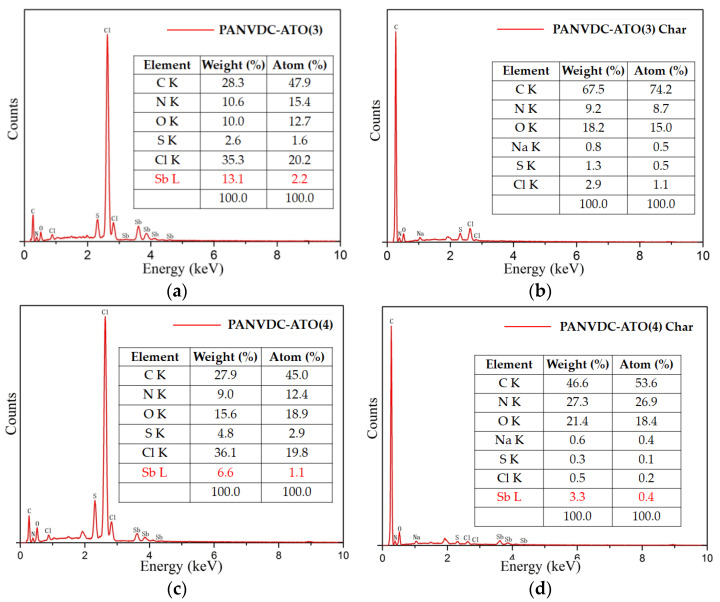
SEM-EDX analysis of the char layers before/after the combustion of PANVDC films containing antimony oxide; (**a**) before combustion of the ATO(3) film, after combustion of the (**b**) ATO(3) char, (**c**) ATO(4) film, and after combustion of the (**d**) ATO(4) char.

Figure 4 presents the SEM-EDX results of the char layers before/after the combustion of PANVDC films containing antimony oxide. As shown in Figure 4a,c, before the combustion of the ATO(3)- and ATO(4)-added films in PANVDC, antimony oxide was observed on the sample surfaces. After combustion, there was no ATO(3) in the PANVDC-ATO(3) char, and a smaller amount of ATO(4) remained in the PANVDC-ATO(4) char (Figure 4b,d). This is because ATO and HCl act mainly as flame retardants in the gas phase. In addition, Sb was detected on the surface of the PANVDC-ATO(4) because ATO(4) has a higher heat resistance after combustion.

### 3.2. Flame Retardancy of PANVDC Films with Flame Retardants

Table 2 lists the flame retardancy of PANVDC films with antimony oxides. This study was conducted using two methods: UL-94 and LOI of the vertical burning test to evaluate the relative flammability and the melt dripping of polymeric materials. 

**Figure 5:**
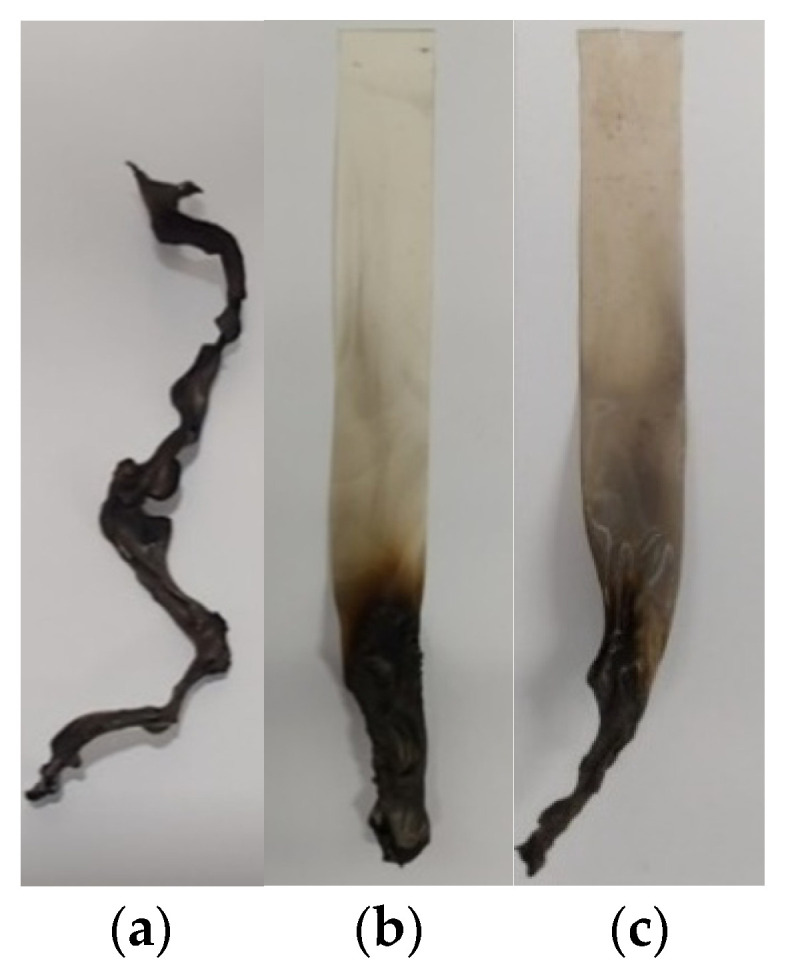
Images of the PANVDC Polymers after UL-94 tests: (**a**) Pure PANVDC; (**b**) PANVDC-ATO(3), (**c**) PANVDC-ATO(4).

Table 2 and Figure 5 present the UL 94 testing result and photographs of samples after the combustion process, which lasted 10 s. Pure PANVDC was burned immediately when exposed to the flame, and extinguished itself when the flame was removed. As shown in Figure 5a, the PANVDC was totally burned and blackened by smoke during burning. On the other hand, when ATO(3) or ATO(4) were added to PANVDC films, approximately 30% of the sample was burned; hence, both samples presented a V-0 rating in the UL94 test. Thus, flame retardancy could be improved by adding antimony oxide to PANVDC.

Based on the above result, the LOI was measured to compare the relative flammability of both PANVDC-ATO(3) and PANVDC-ATO(4). As shown in Table 2, the LOI of pure PANVDC, PANVDC-ATO(3), and PANVDC-ATO(4) films were 26.4%, 29.0%, and 31.2%, respectively. The LOI values of the antimony oxide-added PANVDC were higher than pure PANVDC film. In addition, when comparing the types of antimony oxides, the LOI of PANVDC-ATO(4) was 31.2%, higher than that of PANVDC-ATO(3), which was 29.0%. This results indicate that ATO(4) exhibited higher flame retardancy efficiency because it has a high heat resistance owing to its stable structure and properties. 

### 3.3. Morphology and Mechanical Properties of the PANVDC Fibers with Flame Retardants

**Figure 6:**
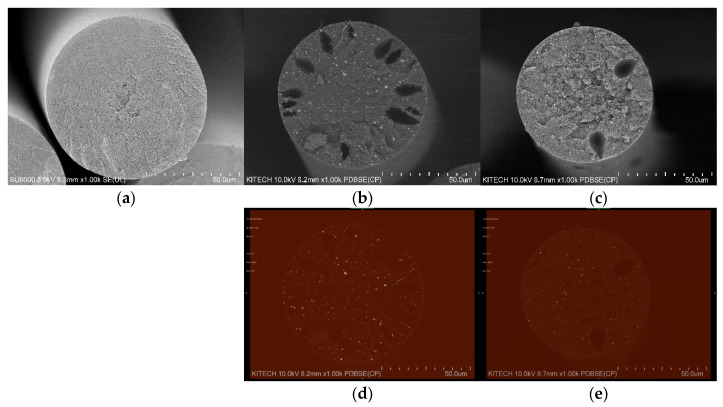
FE-SEM images of PANVDC fibers: (**a**) Pure PANVDC, (**b**) PANVDC-ATO(3), (c) PANVDC-ATO(4) and images of distribution of (**d**)ATO(3) and (**e**) ATO(4) in PANVDC fiber.

Figure 6 shows the cross-sectional SEM images of the wet-spun fibers with ATO(3) and ATO(4). As shown in Figure 6, the PANVDC fiber and PANVDC fiber with the flame retardant had a round cross-section. In addition, no pores were observed in the PANVDC fiber, but several pores were presented in PANVDC-ATO(3) and PANVDC-ATO(4). Hence, pores generated as antimony oxide particles were added to pure PANVDC by interfering with solvent diffusion into/out of the fiber in the coagulation bath during wet spinning [15,16]. 

**Figure 7:**
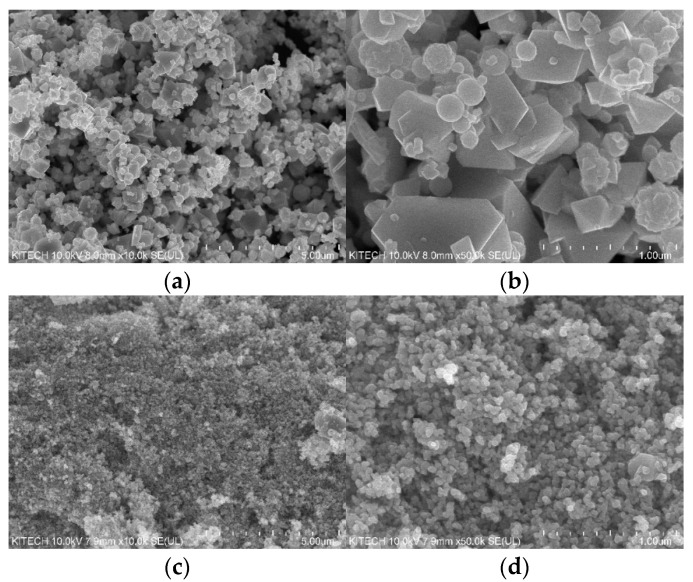
FE-SEM images of the powder particles of (**a**) ATO(3) ×10,000, (**b**) ATO(3) ×50,000, (**c**) ATO(4) ×10,000, and (**d**) ATO(4) ×50,000.

Figure 7 shows the FE-SEM images of the ATO(3) and ATO(4) powder particles. As shown in Figure 7, the ATO(4) particles were smaller than the ATO(3) particles at the same magnification, which affected the formation of pores in the PAN fiber, and the number of pores of PANVDC-ATO(3) was greater than that of PANVDC-ATO(4). It is confirmed that the generation of pores increases as it acts as a factor preventing the solvent inside the fiber from spreading into the coagulation bath after spinning, since antimony trioxide has a relatively larger particle size than antimony tetroxide. In addition, as shown in Figure 6, the white dots observed in the SEM images of PANVDC-ATO(3) and PANVDC-ATO(4) are antimony oxide, showing that the flame retardant is introduced evenly at the cross-section of fibers. Therefore, the flame retardant barely eluted under the coagulation bath conditions during wet spinning and was well introduced into the fiber, ensuring flame retardancy.

**Figure 8:**
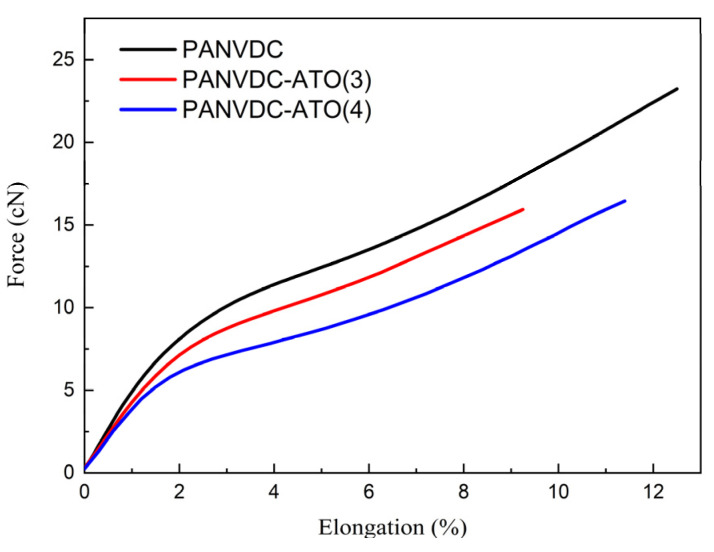
Average force/elongation curve of the drawn PANVDC fibers with the flame retardants.

Table 3 and Figure 8 present the mechanical properties of PANVDC fibers with two types of antimony oxides. The three types of PANVDC fibers were washed and drawn in hot water baths in a continuous process to remove the excess solvent and increase the orientation of the PANVDC chains. As shown in Table 3, the tenacity values of the pure PANVDC, PANVDC-ATO(3), and PANVDC-ATO(4) were 4.42 ± 0.25 g/den, 3.11 ± 0.41 g/den, and 3.73 ± 0.16 g/den, respectively. In the case of elongation, pure PANVDC, PANVDC-ATO(3), and PANVDC-ATO(4) showed an elongation of 12.52 ± 0.34%, 9.34 ± 1.02%, and 11.32 ± 0.59%, respectively. The PANVDC containing the flame retardants showed a slightly lower tenacity and elongation. As confirmed in the FE-SEM images, both tenacity and elongation decreased due to the effect of the pores formed by the addition of antimony oxide. The strength and elongation of PANVDC-ATO(4) were superior to those of PANVDC-ATO(3). The powder particle size of ATO(4) was relatively smaller than ATO(3), it was confirmed that relatively smaller particles were distributed in PANVDC-ATO(4) than in PANVDC-ATO(3), and there was a lower number of pores in the cross-sectional image of the fiber. Thus, PANVDC-ATO(4) exhibited superior mechanical properties compared to PANVDC-ATO(3). 

### 3.4. Thermal Properties of the PANVDC Fibers with the Flame Retardants

**Figure 9:**
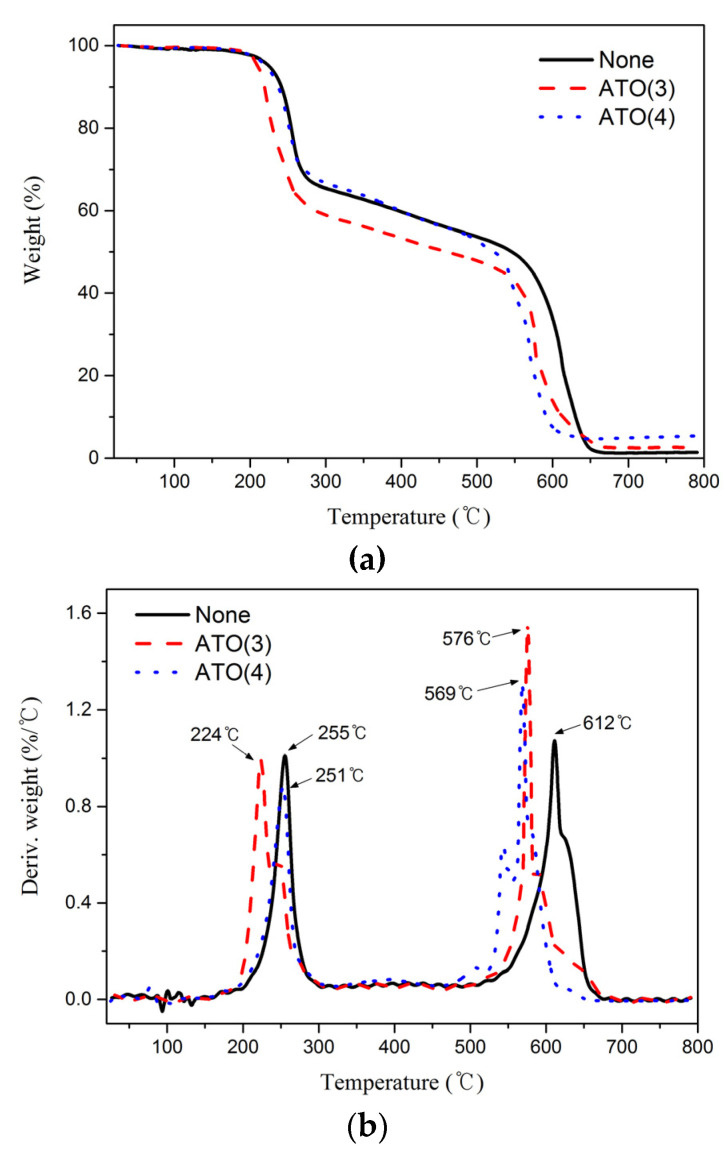
(**a**) TGA and (**b**) DTG curves of PANVDC fibers with antimony oxides at a heating rate 20 °C/min to 800 °C under air conditions.

Figure 9 and Table 4 present the TGA and derivative thermogravimetry (DTG) curves of the PANVDC fibers with antimony oxides. PANVDC showed the two main stages at around 250 °C and 580 °C (Figure 9). 

The first stage was indicated to result from dehydrochlorination and the subsequent reactions generated by HCl. The TMR1 of the PANVDC fibers with antimony trioxide was approximately 30 °C lower than that of PANVDC. On the other hand, PANVDC fibers with antimony tetroxide showed a similar TMR1 to PANVDC (255 °C). At that time, the mass loss of PANVDC-ATO(3) was largest among the fibers, whereas that of PANVDC-ATO(4) was the smallest. Hence, the different properties of materials play an important role in the flame retardant effect of ATO(3) and ATO(4). Thus, ATO(4) has higher heat resistance properties than ATO(3). 

The second-stage weight loss appeared to be caused by the decomposition of the char layer produced by the subsequent reaction after dehydrochlorination [16]. The TMR2 values of PANVDC, PANVDC-ATO(3), and PANVDC-ATO(4) were 612 °C, 576 °C, and 569 °C, respectively. The TMR2 was decreased when antimony oxide was added into PANVDC. This phenomenon is quite different from the first stage. On the other hand, the mass loss of PANVDC-ATO(4) (52%) was lower than PANVDC (64%) and PANVDC-ATO(3) (56%). Only approximately 5% of the residue remained at 800 °C of PANVDC-ATO(4). As confirmed by SEM-EDX, heat resistance was improved because ATO(4), which has an excellent heat resistance and remains on the surface, unlike the other two samples that were completely burned. 

### 3.5. Migration of Antimony from PANVDC Fibers with the Flame Retardants

The harmfulness of PANVDC fibers that contain antimony oxide flame retardants to the human body was assessed by conducting antimony dissolution tests in alkaline and acidic solutions at pH 8 and pH 1.2, respectively. The results are shown in Table 5. 

In general, the antimony dissolution limit prescribed in each standard is 100 mg/kg in an alkaline solution and 60 mg/kg in an acidic solution. As a result of the antimony dissolution test, the amount of antimony elution from the PANVDC fibers introduced with Sb_2_O_3_ or Sb_2_O_4_ in an alkaline solution were all 60 mg/kg, which was detected below the acceptable standard. In the acidic solution, however, PANVDC-ATO(3) and PANVDC-ATO(4) were 114 mg/kg and 60 mg/kg, respectively. Hence, the PANVDC fibers containing ATO(3) had an elution amount over the reference value. Overall, Sb_2_O_4_ could replace Sb_2_O_3_ as a flame retardant in PANVDC fibers for high flame retardancy and safety.

## 4. Conclusions

This study aimed to find an alternative flame retardant to ATO(3). ATO(4) was selected because it has a higher thermal stability and acid and alkali resistance than ATO(3). 

To confirm the possibility of the substitution of ATO(3), which is a representative flame retardant, ATO(4) was selected, and ATO(3)- or ATO(4)-added PANVDC film and wet-spun fibers were fabricated. The flame retardancy was assessed by preparing a film. Both PANVDC-ATO(3) and PANVDC-ATO(4) passed the V-0 rating in UL-94. PANVDC-ATO(4) showed the highest LOI value of 31.2%. 

PANVDC fibers with antimony oxide were fabricated. The morphology, mechanical properties, and thermogravimetric analysis showed that the mechanical and thermal properties of PANVDC fibers with ATO(4) were superior to the PANVDC fiber with ATO(3). In addition, the migration of antimony analysis showed that the elution amount from PANVDC fibers with ATO(4) was below the acceptable standard. 

Based on these results, ATO(4) can be used as an alternative flame retardant to ATO(3) in PANVDC fibers because of its high flame retardancy and safety in humans. 

## Figures and Tables

**Table 1:** Gas products through the pyrolysis of PANVDC fibers containing antimony oxide.

PANVDC		PANVDC-ATO(3)		PANVDC-ATO(4)	
Retention Time (Min)	Pyrolysis Products	Retention Time (Min)	Pyrolysis Products	Retention Time (Min)	Pyrolysis Products
1.469	hydrogen chloride *	1.469	hydrogen chloride *	1.770	hydrogen chloride *
1.679	Acrylonitrile *	1.679	Acrylonitrile *	2.017	Acrylonitrile *
1.908	Methylacrylonitrile *	1.908	Methylacrylonitrile *	2.264	Methylacrylonitrile *
2.923	2,4-pentadienenitrile	2.923	2,4-pentadienenitrile	3.380	2,4-pentadienenitrile
4.313	cyanopentadiene	4.313	cyanopentadiene	-	-
5.319~5.466	chloropyridine isomers *	5.319~5.466	chloropyridine isomers *	-	-
5.694	2-pentenedinitrile	5.694	2-pentenedinitrile	6.225	2-pentenedinitrile
7.404	2-methylenepentanedinitrile *	7.404	2-methylenepentanedinitrile *	7.935	2-methylenepentanedinitrile
7.651	2-methylpentanedinitrile	7.651	2-methylpentanedinitrile	8.054	2-methylpentanedinitrile
7.917	3-methylbenzonitrile	7.917	3-methylbenzonitrile	-	-
8.529	3-chlorobenzonitrile	8.529	3-chlorobenzonitrile	-	-
-	-	9.856	Antimony compound	10.057	Antimony compound
10.551	isophthalonitrile	10.551	isophthalonitrile	11.090	isophthalonitrile
13.935	hexane-1,3,5-tricarbonitrile *	13.935	hexane-1,3,5-tricarbonitrile *	14.456	hexane-1,3,5-tricarbonitrile *
14.282	pentane-1,3,5-tricarbonitrile	14.282	pentane-1,3,5-tricarbonitrile	14.794	pentane-1,3,5-tricarbonitrile
15.389	hexane-1,3-5-tricarbonitrile	15.389	hexane-1,3-5-tricarbonitrile	15.910	hexane-1,3-5-tricarbonitrile

* Major peak.

**Table 2:** Flame retardancy of the PANVDC films with antimony oxides.

Sample	UL-94	LOI (%)
Pure PANVDC	- ^a^	26.4
PANVDC-ATO(3)	V-0	29.0
PANVDC-ATO(4)	V-0	31.2

^a^ Completely burned in the first burning.

**Table 3:** Mechanical properties of PANVDC fibers with antimony oxides.

Sample	Tenacity (g/Den)	Fineness (Denier)	Elongation (%)
Pure PANVDC	4.42 ± 0.25	5.39 ± 0.44	12.52 ± 0.34
PANVDC-ATO(3)	3.11 ± 0.41	5.31 ± 0.45	9.34 ± 1.02
PANVDC-ATO(4)	3.73 ± 0.16	4.29 ± 0.20	11.32 ± 0.59

**Table 4:** TGA and DTG results of the PANVDC fibers with antimony oxides.

Sample	First Stage		Second Stage	
	TMR1 * (°C)	Mass Loss (%)	TMR2 * (°C)	Mass Loss (%)
Pure PANVDC	255	35	612	64
PANVDC-ATO(3)	224	41	576	56
PANVDC-ATO(4)	251	32	569	52

* TMR1 and TMR2: Temperature at the maximum rate of mass loss (%/°C) in the first and second stages, respectively.

**Table 5:** Migration of antimony oxide of PANVDC fibers with antimony oxides.

Sample	Sb Element (mg/kg)	
	Alkaline Solution (pH 8)	Acidic Solutions (pH 1.2)
PANVDC-ATO(3)	60	114
PANVDC-ATO(4)	60	60

## Data Availability

The data presented in this study are available in this article.
